# The quality of sleep: evaluation among university students

**DOI:** 10.3389/fpubh.2023.1270426

**Published:** 2024-01-08

**Authors:** Silvia Angelillo, Vincenza Sansone, Giovanna Paduano, Ludovica Lateano, Gabriella Di Giuseppe, Carmelo Giuseppe Angelo Nobile

**Affiliations:** ^1^Department of Health Sciences, University of Catanzaro “Magna Gracia”, Catanzaro, Italy; ^2^Department of Experimental Medicine, University of Campania “Luigi Vanvitelli”, Naples, Italy; ^3^Department of Pharmacy, Health and Nutritional Sciences, University of Calabria, Arcavacata of Rende, Cosenza, Italy

**Keywords:** quality sleep, students, Covid-19, mental health, survey

## Abstract

**Introduction:**

This study explored the quality of sleep among university students in the South of Italy during the Covid-19 pandemic.

**Methods:**

A cross-sectional study was conducted between March 2022 and January 2023 and involved students over the age of 18, who were invited to complete a self-administered questionnaire using an online application.

**Results:**

Overall, 88% of men and 94.5% of women had Pittsburgh Sleep Quality Index (PSQI) scores of ≥5 and a mean PSQI score of 9.2 ± 3. Students with severe or extremely depression score, with sever or extremely stress score, male and who did not had Covid-19 infection were more likely to have a PSQI global score. Moreover, 62.6% of the students declared a reduction in social relations and 72.3% an increase in the use of social media during the pandemic period. The majority of respondents reported an extremely severe level of depression (68.1%), anxiety (84.4%) and stress (71.9%).

**Conclusion:**

This finding indicate that a relevant percentage of students are poor sleepers with a higher overall PSQI score with depression and stress and underline the role the implementation of public health interventions to promote healthy life styles and in particular focus on the duration of long night sleep.

## 1 Introduction

It is well established that sleep is regulated by a set of physiologic and genetic processes, nonetheless many sleep behaviors are influenced by social, subjective and environmental factors. Generally, sleep occupies 20–40% of people’s days and good sleep patterns are markers of a well-off social status (1, 2).

Sleep disorders have a high prevalence in the general population (3) and have been found to be associated with obesity, weight disorders and metabolic syndrome (4, 5). Nonetheless there is evidence that people are not conscious and aware of the impact of these problems in daily routine (6). Quality of sleep, indeed, is associated with mental disorders and, together, these conditions are public health challenges because both of them have an important impact on individual and global wellness (6–9). Several studies have shown an association between inadequate sleep and frequent mental disorders and lack of sleep negatively affects health (10, 11). In particular, sleep quality and mental disorders have a bidirectional relationship over the life course (12), indeed, poor sleep quality is described as a significant predictor for the onset of depression, anxiety, alcohol abuse and psychosis (13, 14).

Although research most commonly focuses on the associations between insomnia and depression and anxiety, there is also evidence that problems of sleeping are associated with a variety of mental disorders. For example, poor sleep has also been associated with posttraumatic stress or eating disorders (15, 16). Relationship between sleep and mental disorder represents a potentially modifiable risk factor instead of a symptom–disease relationship (9).

In recent years, Covid-19 has changed sleep patterns, representing a source of stress (17, 18). The global effects of these factors are still unknown, on the one hand Covid-19 seems to have had negative effects on the sleep health, while conversely others have benefited of increased sun exposure and increased sleep during the pandemic (19–21). At the same time, quarantine reduced the daylight time for some people, influencing the synchronization of the circadian body clock, consequently worsening many people’s sleep and mood (22, 23). Moreover, during the first waves of pandemic, poor knowledge about Covid-19 has been associated with poor quality of sleep (24).

The determinants of sleep quality, including socio-demographic characteristics, behavioral and lifestyle factors, and mental health status had been largely explored in the literature, especially among university students (25, 26). To our knowledge, no studies had been conducted on the quality of sleep and mental disorders among university students from Southern of Italy during the Covid-19 pandemic. To address this research gap, the Theory of Unpleasant Symptoms (TOUS) has been employed (27). Following the TOUS, indeed, three categories of factors can influence the quality of sleep: physiological, psychological, and situational factors. The research question driving the present study is if quality of sleep of university students has been influenced by Covid-19 pandemic (situational factor), anxiety, stress and depression symptoms (psychological factors), and sleep latency, duration, efficiency and disturbance (physiological factors). Therefore, the aim of this study is to evaluate determinants of sleep quality on university students from Southern Italy in the context of the Covid-19 pandemic.

## 2 Materials and methods

### 2.1 Study population, sampling procedure and data collection

A cross-sectional study was conducted between March 2022 and January 2023 and involved students over the age of 18 enrolled at the Magna Graecia University of Catanzaro.

The required sample size was calculated before the beginning of the study, considering a 95% confidence interval, an alpha error of 5%, a response rate 50% and, since previous data were not available, poor sleep quality was assumed in 50% of students (28). Therefore, the minimum sample size was estimated to be 385 participants.

Data were collected anonymously using a self-administered online questionnaire using the Google Forms® online application. Each student was sent the questionnaire link via institutional email, which contained a brief summary of the study’s objectives, so that participants could decide whether to participate or not, and provide informed consent to participate in the survey. Only those who expressed their consent to participate in the survey and completed the questionnaire in all its parts have been included, since the questionnaire allowed to proceed only by completing all the questions before the final transmission. Furthermore, the questionnaire could be sent only once, in order to minimize the potential for repeated responses. Participants did not receive any form of payment or incentive for participating in this study. The study protocol was approved by the Ethics Committee of the Magna Graecia University of Catanzaro (Protocol n.107, 21 April 2022) and was conducted in accordance with the Declaration of Helsinki.

### 2.2 Survey instrument

The questions were grouped into five sections: (1) socio-demographic characteristics of students; (2) assessment of knowledge on Covid-19 infection and prevention measures; (3) information on the contagion from Covid-19; (4) assessment of sleep quality; (5) assessment of the level of anxiety, stress and depression.

The variables examined in the socio-demographic section included: gender, age, marital status, level of education, occupation, general information on family and home (having or not children, number of people in the household, size of the house, presence/absence of garden or balcony) and information on any changes to the occupation of participants during the Covid-19 pandemic (office work or smart working, face-to-face or distance learning).

Knowledge about Covid-19 infection was assessed through four questions regarding transmission routes and prevention measures. Collection of data on participants regarding the contagion from Covid-19 included having contracted or not Covid-19, having been forced or not to remain in a mandatory quarantine condition, having had close people who tested positive or not, having lost someone close due to Covid-19 eventual, effects that the emergency had had on their social relationships (decrease/improvement of social contacts and use of social media).

Sleep quality was assessed using the 19-item Pittsburgh Sleep Quality Index (PSQI) (29, 30). The PSQI is a self-administered questionnaire that evaluates the quality of sleep with questions about sleep in the previous month. The scale is made up of the following 7 components: subjective sleep quality (from *Very good* = 0 to *Very bad* = 3), sleep latency (sum of two questions describing time to fall asleep, each from 0 to 3), sleep duration (with a score from 0= >7 h to 3 = <5 h), routine sleep efficiency (calculated as (*hours slept/h in bed*) × 100%, with a score from 0= >85% to 3 = <65%), sleep disturbances (sum of nine questions, each with a score from 0 to 3, investigating trouble sleeping), use of sleep medications (from *Not during past month* = 0 to *Three or more times a week* = 3) and daytime dysfunctions (sum of two questions investigating staying awake and enthusiastic during the day, each from 0 to 3). By adding the scores of each section the final score between 0 and 21 is obtained with higher scores indicating a lower quality of sleep. A score ≥ 5 indicates a poor sleep, while <5 a good sleep.

The shortened version of the Depression Anxiety Stress Scales (DASS-21) was used to assess anxiety, stress, and depression (28). The DASS-21 is a self-rating scale in which participants rate the frequency and severity of anxiety, stress, and depression. Depression is investigated by assessing dysphoria, anhedonia, lack of incentives, and low self-esteem; anxiety by somatic symptoms of physiological over-arousal and fear response; stress by irritability, impatience, tension, and arousal levels. Therefore, we will have: low positive affect (DASS-Depression), physiological hyper-activation (DASS-Anxiety) and negative affect (DASS-Stress). Subscale scores are calculated as the sum of responses to the seven items of each subscale and multiplied by 2 to satisfy the 42 items of the original scale (29). The cut-offs for defining moderate levels of depression, anxiety, and stress are ≥14, ≥10, and ≥ 19, respectively (31, 32).

### 2.3 Statistical analysis

Data were analyzed with descriptive (proportions, means, standard deviations) and inferential (bivariate and multivariate analysis) statistics using the Stata software version 15.1 (33). First, descriptive statistics were conducted to summarize the main characteristic of the sample. Second, a univariate analysis was performed using the chi-square test, student’s *t*-test and Fisher’s exact test to evaluate the association between several potential determinants and being a poor or good sleeper (poor sleeper = 0; good sleeper = 1) (Table 1). All independent variables considered potential determinants of quality of sleep (continuous) were introduced in the multivariate linear regression model constructed to identify factors associated with this outcome.

**Table 1:** Comparing poor and good sleepers according to socio-demographic characteristics, knowledge about Covid-19 transmission, Covid-19 related data and mental health.

Characteristics	Total (n: 473)		Poor sleeper (n: 439, 92.8%)		Good sleeper (n: 34, 7.2%)	
Socio-demographic characteristics	N	%	N	%	N	%
Gender						
Male	125	26.4	110	88	15	12
Female	348	73.6	329	94.5	19	5.5
			*χ*^2^ = 5.9, df = 1, *p* = 0.015			
Age group, (years)	24.4 ± 5.25 (range:19–63)^*^					
	*t*-test (471) = −0.22 *p* = 0.83					
19–25	324	73.3	320	93.6	22	6.4
≥26	131	27.7	119	90.8	12	9.2
			*χ*^2^ = 1.06, df = 1, *p* = 0.304			
Marital status						
Unmarried/widowed /divorced	353	74.6	325	92.1	28	7.9
Married/cohabitant	120	25.4	114	95	6	5
			*χ*^2^ = 1.15, df = 1, *p* = 0.283			
Occupation						
Students	449	94.9	417	92.9	32	7.1
Working students	24	5.1	22	91.7	2	8.3
			*F*-test = 0.69, df = 1			
Education level						
High school	403	85.2	373	92.6	30	7.4
University degree/master	70	14.8	66	94.3	4	5.7
			*χ*^2^ = 0.27, df = 1, *p* = 0.605			
Smart working/Distance learning						
No	15	3.2	15	100	0	0.0
Yes	458	96.8	424	92.6	34	7.4
			*F*-test = 0.62, df = 1			
Number of cohabitants, (ordinal)	2.73 ± 1.04 (range:1–8)^*^					
	*t*-test (471) = −0.02 *p* = 0.98					
1	59	12.5	57	96.6	2	3.4
2	126	26.6	115	91.3	11	8.7
≥3	288	60.9	267	92.7	21	7.3
			*χ*^2^ = 1.73, df = 2, *p* = 0.421			
Home size (sq.m.)						
≤80	64	13.5	58	90.6	6	9.4
80–150	271	57.3	258	95.2	13	4.8
>150	138	29.2	123	89.1	15	10.9
			*χ*^2^ = 5.58, df = 2, *p* = 0.061			
Having a garden or balcony						
No	27	5.7	26	96.3	413	92.6
Yes	446	94.3	1	3.7	33	7.4
			*χ*^2^ = 0.52, df = 1, *p* = 0.470			
Knowledge about Covid-19 transmission	N	%	N	%	N	%
Covid-19 is transmitted through coughing/sneezing						
False	15	3.2	14	93.3	1	6.7
True	458	96.8	425	92.8	33	7.2
			*F*-test = 1, df = 1			
Social distancing reduces the risk of contracting Covid-19						
False	5	1.1	4	80	1	20
True	468	98.9	435	92.9	33	7.1
			*F*-test = 0.31, df = 1			
The mask is useful to prevent the Covid-19						
False	7	1.5	6	85.7	1	14.3
True	466	98.5	433	92.9	33	7.1
			*F*-test = 0.41, df = 1			
The exchange of the air in the room reduces the risk of contracting Covid-19						
False	15	3.2	15	100	0	0.0
True	458	96.8	424	92.6	34	7.4
			*F*-test = 0.62, df = 1			
Covid-19 related data						
Covid-19 positive						
No	261	55.2	240	91.9	21	8.1
Yes	212	44.8	199	93.9	13	6.1
			*χ*^2^ = 0.64, df = 1, *p* = 0.423			
Time of Covid-19 infection*^a^*						
First and second waves	14	6.6	12	85.7	2	14.3
Third wave	20	9.4	18	90	2	10
Subsequent waves	178	84	169	94.9	9	5.1
			*χ*^2^ = 2.5, df = 2, *p* = 0.287			
Someone close positive to Covid-19						
No	100	21.1	88	88	12	12
Yes	373	78.9	351	94.1	22	5.9
			*χ*^2^ = 4.4, df = 1, *p* = 0.036			
Someone close died of Covid-19						
No	430	90.9	398	92.6	32	7.4
Yes	43	9.1	41	95.3	2	4.7
			*F*-test = 0.76, df = 1			
Forced quarantine						
No	167	35.3	150	89.8	17	10.2
Yes	306	64.7	289	94.4	17	5.6
			*χ*^2^ = 3.46, df = 1, *p =* 0.063			
Changes in social relationship during the pandemic						
Decreased	296	62.6	275	92.9	21	7.1
Stable	166	35.1	153	92.2	13	7.8
Improved	11	2.3	11	100	0	0.0
			*χ*^2^ = 0.96, df = 2, *p =* 0.619			
Changes in social media use during the pandemic						
Decreased	11	2.3	10	90.9	1	9.1
Stable	120	25.4	110	91.7	10	8.3
Improved	342	72.3	319	93.3	23	6.7
			*χ*^2^ = 0.41, df = 2, *p* = 0.816			
Mental health						
Depression						
Normal	-	-	-	-	-	-
Mild	-	-	-	-	-	-
Moderate	82	17.3	71	86.6	11	13.4
Severe	69	14.6	61	88.4	8	11.6
Extremely severe	322	68.1	307	95.3	15	4.7
			*χ*^2^ = 9.86, df = 2, *p* = 0.007			
Anxiety						
Normal	-	-	-	-	-	-
Mild	-	-	-	-	-	-
Moderate	12	2.5	9	75	3	25
Severe	62	13.1	51	82.3	11	17.7
Extremely severe	399	84.4	379	95	20	5
			*χ*^2^ = 18.89, df = 2, *p* < 0.001			
Stress						
Normal	3	0.6	3	100	0	0.0
Mild	13	2.7	10	76.9	3	23.1
Moderate	34	7.2	27	79.4	7	20.6
Severe	83	17.5	72	86.7	11	13.3
Extremely severe	340	71.9	327	96.2	13	3.8
			*χ*^2^ = 24.65, df = 4, *p* < 0.001			

^*^Mean ± Standard Deviation (Range).

^a^ Only among those who had Covid-19 infection.

In the multivariate linear model the following independent variables, which were judged to potentially have influence on the above mentioned outcome (Model), were included: gender (male = 0, female = 1); age in years (continuous); occupation (students = 1, working students = 2); smart working/distance learning (no = 0,yes = 1); Covid-19 infection (no = 0,yes = 1); anxiety score

(normal/mild/moderate = 0; severe/extremely severe = 1); depression score (normal/mild/moderate = 0;severe/extremely severe = 1); stress score (normal/mild/moderate = 0; severe/extremely severe = 1).

Backward stepwise procedures were applied, including in the final models only the characteristics that provided a significant explanation of the outcomes, with a threshold of *p*-values of 0.2 for entering and of 0.4 for being retained. Standardized regression coefficients (β) were presented. All analyses were two-sided and the level of statistical significance was set at *p* equal to or less than 0.05.

## 3 Results

Of the 930 university students invited, 473 agreed to participate in the survey, with a response rate of 50.9%. The main characteristics of the examined sample are reported in Table 1. A large majority of participants were women (73.6%), with a mean age of 24.4 (range = 19–63 ± 5.25), and 74.6% were unmarried, widowed or divorced. Among the respondents, 14.8% had already another degree, 5.1% had a working activity and, the majority, during the pandemic, attended distance learning (96.8%). Moreover, 62.6% of the students declared a reduction in social activities and 72.3% an increase in the use of social media. 60.9% of respondents had more than two cohabitants, and most of respondents (94.3%) had an outdoor space (garden or balcony). Quarantine was imposed on 64.7% of them, and 78.9% of respondents had someone close who was positive to Covid-19. In addition, 9.1% had experience of someone close who died for Covid-19 infection. 44.8% of participants had the Covid-19 infection, the majority of them (84%) after the third wave. The investigation of students’ knowledge showed that almost all participants were aware that Covid-19 transmission occurs through coughing or sneezing, that it is reduced by air exchange (96.8%), and that social distancing and masks prevent Covid-19 infection (>98%). The majority of respondents reported an extremely severe level of depression (68.1%), anxiety (84.4%) and stress (71.9%).

Analyzing the quality of sleep of students, it emerged that 88% of men and 94.5% of women had PSQI scores ≥5 and a mean PSQI score of 9.2 ± 3. Tables 1, 2 highlight the results of the univariate and multivariate analyses of the outcome of interest. Univariate analysis shows significant differences according to several characteristics. In particular, PSQI was higher in women, in those who had someone close who tested positive for Covid-19, in students who had forced quarantine, and among subjects with extremely severe levels of depression, anxiety and stress (Table 1).

**Table 2:** Linear regression model investigating determinants of sleep quality.

Variable	Coeff	SE	*t*	*p*
Model. Pittsburgh Sleep Quality Index (PSQI) global score				
*F*(6, 466) = 10.87; R^2^ = 12%; adjusted R^2^ = 11% *p* < 0.001				
Gender (male = 0; female = 1)	0.71	0.3	2.39	0.017
Covid-19 positive (no = 0; yes = 1)	0.56	0.26	2.17	0.031
Depression score (normal/mild/moderate = 0; severe/ extremely/severe = 1)	1.17	0.41	2.85	0.004
Stress score (normal/mild/moderate = 0; severe/ extremely severe = 1)	1.80	0.51	3.56	<0.001
Age in years, (continuous)	0.29	0.03	1.20	0.231
Smart working (no = 0; yes = 1)	0.86	0.75	1.15	0.249

The following variables were deleted by the backward elimination procedure: occupation and anxiety score.

The results of the multivariate analysis showed that students with severe or extremely severe depression score, with severe or extremely severe stress score, who were females and who had contracted Covid-19 infection were more likely to have a high PSQI global score (Model in Table 2).

As summarized in Table 3, more than half of respondents (54.3%) had very bad subjective sleep quality in the previous month. In particular, more than half of students had difficulty in sleep initiation within 30 min 3 times or more per month (59.2%), and only 34.6% of participants reported long sleep duration lasting >7 h. Almost half of the students perceived a sleep efficiency >85% in the previous month (48.3%). Moreover, almost half of students (49.3%) reported to have experienced sleep disturbances one or two times a week (waking up or going to the bathroom in the middle of the night, not being able to breathe well, coughing or snoring, feeling hot or cold, having bad dreams or pain). Furthermore, 17.5% said they had taken medication as a sleep aid, and one half (47.7%) reported difficulty in staying awake while driving or eating meals or with maintaining their enthusiasm while completing their duties in the previous month.

**Table 3:** Pittsburgh sleep quality index components and factor.

Components	Very good N (%)	Fairly good N (%)	Fairly bad N (%)	Very bad N (%)

1: Subjective sleep quality				
During the past month, how would you rate your sleep quality overall?	24 (5.1)	-	192 (40.6)	257 (54.3)
2: Sleep latency	0	1–2	3–4	5–6
Sum of two response: During the past month, how long (in minutes) has it usually taken you to fall asleep each night and during the past month, how often have you had trouble sleeping because you cannot get to sleep within 30 min	38 (8)	155 (37.8)	157 (33.2)	123 (26)
3: Sleep duration	>7 h	6–7 h	5-6 h	<5 h
During the past month, how many hours of actual sleep did you get at night? (This may be different than the number of hours you spent in bed)	163 (34.6)	270 (57.3)	25 (5.3)	13 (2.8)
4: Sleep efficiency	>85%	75–84%	65–74%	<65%
(Hours slept/ h in bed) × 100% Hour slept: During the past month, how many hours of actual sleep did you get at night? Hours in bed: sum of two response: during the past month, what time have you usually gone to bed at night? and during the past month, what time have you usually gotten up in the morning?	228 (48.3)	131 (27.8)	63 (13.4)	50 (10.6)
5: Sleep disturbance	0	1–9	10–18	19–27
Sum of 9 response: during the past month, how often have you had trouble sleeping because you wake up in the middle of the night or early morning and have get up to use the bathroom and cannot breathe comfortably and cough or snore loudly and feel too cold and feel too hot and have bad dreams and have pain	2 (0.4)	238 (50.3)	208 (44)	25 (5.3)
6: Use of sleep medications	Not during past month	Less than one a week	Once or twice a week	Three or more times a week
During the past month, how often have you taken medicine to help you sleep (prescribed or “over the counter”)?	390 (82.5)	30 (6.3)	26 (5.5)	27 (5.7)
7: Daytime dysfunction	0	1–2	3–4	5–6
Sum of two response: During the past month, how often have you had trouble staying awake while driving, eating meals, or engaging in social activity? And during the past month, how much of a problem has it been for you to keep up enough enthusiasm to get things done?	43 (9.1)	204 (43.1)	170 (35.9)	56 (11.8)

## 4 Discussion

As highlighted by the World Health Organization (34), during the Covid-19 pandemic, there was a potential risk of increase in mental illness. All the worries, resulting from the almost inevitable health risks and social distancing, could have had a major impact on daily functioning and nighttime sleep. These scenarios impacted individuals’ daily routines, emotional wellbeing, sleep quality and mental health, leading to changes in sleep patterns, representing a source of stress. The present study provides results on the sleep quality of students during the pandemic and on several factors that can affect the quality of sleep.

Almost all participants demonstrated an optimal knowledge about the transmission of Covid-19. Indeed, data collection was conducted in 2022–2023, when the major waves of the pandemic were over in Italy and people were well informed on how to avoid infection. Although strong knowledge about Covid-19 has been associated with less psychological distress and with better quality of sleep during the first waves of pandemic (24, 35), reported findings are not consistent with previous studies since no association has been reported between knowledge on Covid-19 and sleep quality or psychological distress in the current epidemiological context. It has been observed that more than half of students had difficulty in sleep initiation and only one third reported long sleep duration (lasting >7 h), particularly in women. These results are similar to those reported in college students in USA, respectively 48 and 36.7% (36). Indeed, almost half of our students declared to have experienced sleep disturbances and this frequency is higher than that reported in a similar population (30%) (36). One half of participants declared to have experienced difficulty in staying awake while driving or eating meals or with maintaining their enthusiasm while completing their duties in the previous month, and even this sleep component is in contrast with what reported (25%) in a similar group (36).

The prevalence of poor sleepers in our population (92.8%) is higher than that reported in two similar Italian studies (around 75%) (37, 38) and significantly higher than that reported in German (36.9%) (39) and Chinese students (66.2%) (40).

In our study the mean PSQI global score (9.2 ± 3) is significantly higher in comparison with similar studies that used the same scale to evaluate the quality of sleep during lockdown in Italian students (PSQI mean score from 5.8 to 6.7) (41–43) and in USA students (PSQI mean score around 7.5) (44, 45).

In addition to the interpretation of the PSQI total score, the detailing of results on the domains of this instrument is worth discussing. In our study the mean of sleep latency is 1.8 ± 0.92 which is similar to the results reported in the same period by Benham et al. (1.49 ± 1) (44), Romero Blanco et al. (1.54 ± 1.06) (46) and Somma et al. (1.49 ± 1) (43); moreover, in our population it was revealed an increase in daytime dysfunction (1.5 ± 0.82) compared to the studies of Viselli et al. and Somma et al. (1.21 ± 8 and 1.24 ± 0.70) (42, 43). Mean sleep disturbance in our study was 1.5 ± 0.6, which is higher in comparison with Romero-Blanco et al. (1.12 ± 0.43) (46) and Somma et al. (1.14 ± 0.45) (43), whereas use of sleep medication is the same reported by Benham et al. (0.3 ± 0.82) (44). In line with the literature (38, 44, 46), college students reported a decrease in sleep duration (0.8 ± 0.67 in our study) and sleep efficiency (0.9 ± 1.01 in our study).

The present study also found a strong association, recently documented also in the Italian population (42, 47), between those who have poor sleep quality and psychological distress. The majority of respondents reported an extremely severe level of depression (68.1%), anxiety (84.4%) and stress (71.9%), in contrast with the study of Najafi Kalyani et al. (48) reporting lower severe level of depression (14.7%), anxiety (32.4%) and stress (24.8%). In the study of Najafi Kalyani et al. (48) a statistically significant relationship was found between the student’s stress levels and sleep quality (*p* < 0.001), which was confirmed by our study. We observed that the change in sleep quality (PSQI global score) was stronger in participants with a high DASS-21 score, similar to previous Italian studies in students and general population, in particular in the South of Italy (41, 49).

Sleep and stress are closely linked, at multiple levels, with current evidence supporting a bidirectional association between sleep and stress (50). Furthermore, the worsening of sleep quality is directly related to the motivation of university students (51).

This study presents some potential limitations that need to be dealt with before interpreting the results. First, the analyses were based on cross-sectional data, and therefore, the nature of the associations limited us from drawing definitive causal conclusions about the observed relationships between determinants and quality of sleep. Second, respondents may have been influenced to give the most “desirable” answers, and this may have produced overestimation of the quality of sleep. Third, this study was conducted after the major waves of the pandemic in Italy and questions were asked to investigate the quality of sleep during the pandemic period, therefore, the time occurred may have had an influence on the recall of sleep quality. Fourth, the majority of the sample was composed by students in Southern Italy from a single university, and our sample might not be completely representative of the Italian students or other populations or age groups such as not students young adults. Fifth, there was potential bias attributable to the use of a self-reporting instrument, recall bias and social desirability bias, and we were unable to accurately reflect the participants’ responses about actual sleep quality or mental health status. Moreover, self-reported data and the use of a cross-sectional design may limit the generalizability of the findings. Additionally, the study did not explore potential confounding factors that could influence sleep quality, such as pre-existing medical conditions, medication use, substance use, or prior sleep disorders. Finally, it was not possible to collect information on students who refused to participate in the study, which may have different characteristics than the study participants. Although those who chose to not participate may have a different quality of sleep or mental health status, which could affect the generalization of results, our response rate was sufficiently high to suggest that no substantial differences in the estimates would have been introduced by the results on non-responders.

In conclusion, a relevant percentage of students are poor sleepers with a higher overall PSQI score associated with depression and stress. Future studies should monitor sleep quality in a wider sample of students involving multiple universities and including additional determinants of sleep quality, such as obesity, current smoking status and binge drinking. The results of this study suggest the implementation of public health interventions to follow-up people with poor quality of sleep and promote healthy life styles focusing on the duration of night sleep.

## Data availability statement

The raw data supporting the conclusions of this article will be made available by the authors, without undue reservation.

## Ethics statement

The studies involving humans were approved by Ethics Committee of the Magna Graecia University of Catanzaro. The studies were conducted in accordance with the local legislation and institutional requirements. The participants provided their written informed consent to participate in this study.

## Author contributions

SA: Conceptualization, Data curation, Investigation, Writing – original draft. VS: Formal analysis, Resources, Writing – original draft. GP: Formal analysis, Resources, Writing – original draft. LL: Data curation, Writing – original draft. GDG: Formal analysis, Writing – original draft. CGAN: Conceptualization, Formal analysis, Methodology, Supervision, Validation, Writing – original draft.
